# Minimum Net Driving Temperature Concept for Membrane Distillation

**DOI:** 10.3390/membranes10050100

**Published:** 2020-05-14

**Authors:** Bastiaan Blankert, Johannes S. Vrouwenvelder, Geert-Jan Witkamp, Noreddine Ghaffour

**Affiliations:** 1 Water Desalination and Reuse Center (WDRC), Biological and Environmental Science and Engineering Division (BESE), King Abdullah University of Science and Technology (KAUST), Thuwal 23955-6900, Saudi Arabia; bastiaan.blankert@kaust.edu.sa (B.B.); geertjan.witkamp@kaust.edu.sa (G.-J.W.); Noreddine.Ghaffour@kaust.edu.sa (N.G.); 2 Department of Biotechnology, Faculty of Applied Sciences, Delft University of Technology, Van der Maasweg 9, 2629 HZ Delft, The Netherlands

**Keywords:** exergy efficiency, thermodynamic analysis, desalination, optimization

## Abstract

In this study, we analyzed the heat requirement of membrane distillation (MD) to investigate the trade-off between the evaporation efficiency and driving force efficiency in a single effect MD system. We found that there exists a non-zero net driving temperature difference that maximizes efficiency. This is the minimum net driving temperature difference necessary for a rational operational strategy because below the minimum net driving temperature, both the productivity and efficiency can be increased by increasing the temperature difference. The minimum net driving temperature has a similar magnitude to the boiling point elevation (~0.5 °C for seawater), and depends on the properties of the membrane and the heat exchanger. The minimum net driving temperature difference concept can be used to understand the occurrence of optimal values of other parameters, such as flux, membrane thickness, and membrane length, if these parameters are varied in a way that consequently varies the net driving temperature difference.

## 1. Introduction

Membrane distillation (MD) is a thermally driven membrane separation process, where a hot saline stream is separated into pure water and a more concentrated brine. A stream of hot feed water and cold pure water are separated by a porous hydrophobic membrane that allows only water vapor to pass. The temperature difference between either side of the membrane causes a vapor pressure gradient that drives the separation process [1]. MD is considered an attractive potential alternative desalination technology because it has lower equipment costs [2], lower pretreatment requirements [3], its utilization of waste heat [3,4], and its opportunities for integration with renewable energy generation [5,6,7]. Moreover, MD is often considered a building block in novel hybrid desalination concepts [8].

MD has currently not yet been successfully commercialized for desalination, despite the potential advantages outlined above. For small pilot and lab-scale systems, heat requirements ranging from 100–2200 kWh/m^3^ have been reported [3,9,10], indicating that the thermal efficiency needs to be improved if the technology is to compete with alternative thermal processes. Furthermore, there are different operating principles, such as direct contact MD (DCMD), vacuum MD (VMD), air gap MD (AGMD), and sweeping gas membrane distillation (SGMD), in addition to different membrane element and staging configurations [1,2,5,11,12]. There is no apparent consensus in the community on which of these basic variants, configurations, membrane properties, and/or operational conditions are optimal. A recent extensive cost analysis of different MD systems suggested that AGMD is favorable compared with DCMD; however, this depends on the valuation of thermal energy, electrical energy, the membrane, and the system price. Furthermore, this favorability also depends on the scale of the system and variant-specific assumptions that must be made [13]. The influence of the configuration and operational settings can be compared in a broad and generalized way, based on a thermodynamical framework analyzing the heat flow. This paper analyzes one aspect within this framework, namely the net driving temperature difference.

Exergy is a concept that allows different forms of energy (e.g., heat, chemical energy, and electricity) to be compared based on the maximum extractable work [14]. The exergy efficiency of a process expresses how well that process can convert the available energy source into work. The concept of exergy efficiency allows desalination processes to be compared in a broader context; for example, thermal desalination vs. electricity generation plus reverse osmosis. For thermal processes, for instance, the exergy content of the utilized heat is proportional to the temperature difference between the heat source and the cooler. Thus, thermal processes can be attractive, despite their seemingly large energy requirement, because the extractable work from a low-grade heat source is small. There is a subtle difference between exergy efficiency and thermal efficiency. For example, by applying local resistive heating [15] or inductive heating [16], the thermal efficiency of a membrane distillation system may be improved. However, the conversion of electricity into heat represents in itself an exergy loss.

The objective of this work is to investigate the trade-off between the evaporation efficiency and the driving force efficiency. The evaporation efficiency is related to the heat flow conducted through the membrane, which does not contribute to the separation process and is thus an exergy loss. The driving force efficiency indicates the exergy used to drive the entire process at the desired production rate. Here, we demonstrate that there is a net driving temperature difference that minimizes the heat requirement. We show that this heat requirement is the minimum net driving temperature difference needed for a rational operating strategy that desires a favorable trade-off between the productivity and heat requirements. Furthermore, we discuss the implications of a minimum net driving temperature difference for the overall exergy efficiency and desired membrane properties, which we investigate by analyzing a single effect MD system.

## 2. Theoretical Background

**Figure 1:**
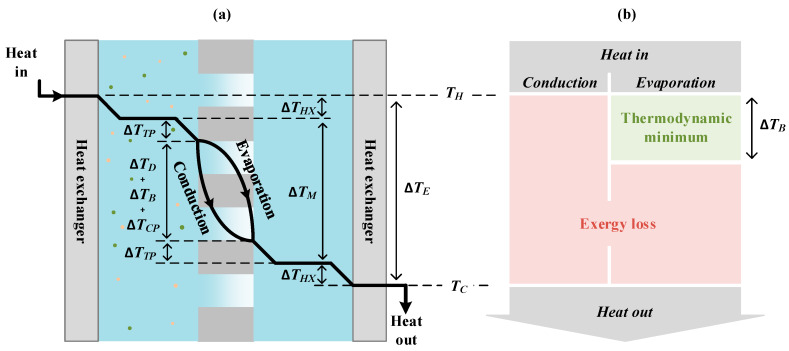
(**a**) Schematic representation of a single effect membrane distillation process. The effect consists of a heat-exchanging surface that transfers heat from the heater to the feed water; a porous hydrophobic membrane that facilitates the vapor transport from the feed to the permeate; and a heat-exchanging surface that transfers the output heat to the cooler. For the analysis of thermal efficiency, it is useful to follow the heat flow and temperature difference that occur as the heat flows through the system. Thus, the following should be monitored: the boundary layer near a heat exchanger Δ*T_HX_*; the temperature difference over the temperature polarization layer Δ*T_TP_*; the temperature difference over the membrane layer consisting of the boiling point elevation Δ*T_B_*; the additional boiling point elevation due to concentration polarization Δ*T_CP_*; and the net driving temperature difference Δ*T_D_*. Through the membrane, the heat is assumed to take two parallel and independent paths, i.e., conduction and evaporation–condensation. (**b**) The total exergy conversion is proportional to the product of the temperature difference and the specific heat flow, as visualized by the green rectangle. Here we assume that the polarization phenomena are identical on both sides of the membrane; however, we could follow the same approach by replacing 2Δ*T_TP_* with Δ*T_TP,F_* + ΔT*_TP,P_*, etc. The thermodynamic minimum follows from the minimum specific heat, i.e., the heat of evaporation and the minimum temperature difference, which is the boiling point elevation.

We based the thermodynamic analysis presented here on following the heat flow through the membrane system, which itself is based on fundamental thermodynamic principles and several assumptions taken from the literature. We illustrate this analysis with a single effect MD system. An effect consists of heating, evaporation, condensation, and cooling, and is the most straightforward system that contains the essential components and phenomena that are relevant for MD. Figure 1 sketches a single effect membrane distillation system where the heater is directly in contact with the feed water, heating the water homogeneously to avoid a temperature gradient in the length direction. The cooler is in contact with the permeate in a similar way. The porous hydrophobic MD membrane separates the feed and the permeate side. The heat is transported through the following layers: the boundary layer near the heater; the ideally mixed feed water bulk; the temperature polarization (TP) layer at the feed side; the membrane; the TP layer at the permeate side; the ideally mixed permeate bulk; and finally, the boundary layer near the cooler. Each of these layers is associated with a temperature difference. The total temperature difference between the two sides of the effect ($\Delta T_{E}$) is the total driving force of the process. The production rate is directly related to the choice of $\Delta T_{E}$.

The exergy input (B) is partially converted into the work of separation and partially destroyed due to inefficiencies. In a thermal process, the exergy input is proportional to the heat (H) and the temperature difference between the heat source ($T_{H}$) and heat sink ($T_{C}$). In the case of a single effect MD, as sketched in Figure 1, the temperature difference between the heat source and heat sink is $\Delta T_{E}$. Thus, the specific exergy input is equivalent to the Carnot formula for the theoretically maximum extractable work by the heat transfer between two reservoirs [14]:

(1) $$B=H\frac{\Delta T_{E}}{T_{H}}$$

The exergy efficiency ($\eta_{II}$) is the ratio of the total exergy input and its thermodynamic minimum ($B_{TD}$), which is equal to the minimum work of separation. In the definition of the exergy input (Equation (1)), two factors could be influenced by the operational settings, namely the specific heat (*H*) and the driving temperature difference (Δ*T_E_*). Hence, the exergy efficiency is considered to be the product of the evaporation efficiency (related to *H*) and the driving force efficiency (related to Δ*T_E_*). In other words, only the losses inside the effect are considered, and external losses, such as pump energies, are not described. The evaporation efficiency ($\eta_{E}$) is the ratio of the specific heat (H) and its thermodynamic minimum, whereas the driving force efficiency is the ratio of the total driving force ($\Delta T_{E}$) and its thermodynamic minimum.

(2) $$\eta_{II}=\frac{B_{TD}}{B}=\eta_{D}\eta_{E}$$

### 2.1. Driving Force Efficiency

Dissolved salt and other solutes affect the thermodynamic properties of the feed water. These colligative thermodynamic properties include osmotic pressure, vapor pressure reduction, and boiling point elevation ($\Delta T_{B}$) [14]. The boiling point elevation is the temperature difference between pure water and saline water, where the vapor is in equilibrium between both. The boiling point elevation is directly related to the minimum work of separation. This value is the thermodynamic minimum of the temperature difference. Thus, the driving force efficiency can be given by

(3) $$\eta_{D}=\frac{\Delta T_{B}}{\Delta T_{E}}$$

### 2.2. Evaporation Efficiency

Figure 1 shows a schematic representation of the heat flow in an MD system. In this figure, the amount of heat that flows is identical at every location and is determined by the phenomena at the membrane interface. Here, we consider that there are two parallel paths, namely conduction and evaporation. We can assume that there is no coupling between the conductive heat transport and convective heat transport via the evaporation heat [17]. The specific heat associated with evaporation ($\Delta H_{v}$) is useful as it contributes to the objective of the process. The heat of evaporation is the thermodynamic minimum of the amount of heat transported from the feed to the permeate because the transport of the water implies evaporation and condensation. The conductive specific heat ($H_{C}$) serves no purpose and is considered a loss. Thus, the evaporation efficiency is defined as [18]

(4) $$\eta_{E}=\frac{\Delta H_{v}}{\Delta H_{v}+H_{C}}$$

The conductive heat flow is proportional to the temperature difference between both interfaces of the membrane and the heat conduction coefficient ($k_{M}$) [19,20,21,22]. The polarization temperature differences $\Delta T_{HX}$ and $\Delta T_{TP}$ occur outside of the membrane and do not directly affect the conductive transmembrane heat flow. As the conductive heat flow is independent of the water flow, the specific conductive heat, or conductive heat per unit of produced permeate, is inversely proportional to the flux $J_{W}$.

(5) $$H_{C}=\frac{k_{M}}{J_{W}}\left(\Delta T_{D}+\Delta T_{B}+\Delta T_{CP}\right)$$

The filtration flux is usually expressed in terms of the vapor pressure difference across the membrane and a permeability coefficient. However, for the analysis of the heat requirement, it is more convenient to work with temperature differences. Following the approach of Schofield et al. [20], the filtration flux is expressed as the product of a temperature-related permeability coefficient $\left(L_{T}\right)$ and the net driving temperature difference between both interfaces of the membrane ($\Delta T_{D}$).

(6) $$J_{W}=L_{T}\Delta T_{D}$$

Note that the permeability coefficient depends on the average temperature in the pore. We assume that a constant average temperature in the pore is maintained by realizing the driving temperature differences while simultaneously modifying the heater and cooler temperature. Under that assumption, the permeability can be treated as a constant. The same assumption is also applied to the heat transfer coefficient of the membrane. Consequently, the evaporation efficiency can be rewritten as follows:

(7) $$\eta_{E}=\frac{1}{1+K_{T}\left(1+\frac{\Delta T_{B}+\Delta T_{CP}}{\Delta T_{D}}\right)}$$

where $K_{T}$ is a membrane parameter that expresses the ratio between the conductive heat transfer resistance and evaporative heat transfer resistance of the membrane, given by

(8) $$K_{T}=\frac{k_{M}}{\Delta H_{v}L_{T}}$$

This membrane parameter has a similarity with a membrane parameter presented by Deshmukh et al. [21], which is the ratio of the membrane heat transfer coefficient and the membrane permeability (defined for vapor pressure difference rather than temperature difference).

### 2.3. Net Driving Temperature Difference Maximizing Thermal Efficiency

The efficiency in a single effect MD system may be given by the product of the evaporation efficiency and the driving force efficiency, which can be written as follows:

(9) $$\eta_{D}\eta_{E}=\left(\frac{\Delta T_{B}}{\Delta T_{B}+\Delta T_{D}+2\Delta T_{TP}+\Delta T_{CP}+2\Delta T_{HX}}\right){\left(1+K_{T}\left(1+\frac{\Delta T_{B}+\Delta T_{CP}}{\Delta T_{D}}\right)\right)}^{-1}$$

An inspection of this equation reveals that the efficiency can be increased by reducing the effect of the polarization phenomena ($\Delta T_{TP}$ and $\Delta T_{CP}$) and by reducing the temperature difference that drives the heat exchangers ($\Delta T_{HX}$), as is well established in the field. For these temperature differences ($\Delta T_{TP},\;\Delta T_{CP},\;\Delta T_{HX}$), the efficiency is maximized by aiming for arbitrarily small values. In contrast, there is a specific (non-zero) value of the net driving temperature difference ($\Delta T_{D}$) that maximizes the efficiency. This can be seen by considering that for large values of $\Delta T_{D}$, the driving force efficiency tends to zero (left term), and for small values of $\Delta T_{D}$, the evaporation efficiency tends to zero (right term). The existence of an optimal driving force is interesting because a maximum thermodynamic efficiency is often achieved when the driving force is infinitesimal in a typical thermodynamic reversible process [14]. What is also noteworthy is that when the inverse applies to the boiling point elevation, i.e., with increasing salinity (Δ*T_B_*), the driving force efficiency increases and the evaporation efficiency decreases. This observation differs from the results presented by Brogioli et al. [23], who showed, by a thermodynamic analysis of conventional thermal desalination (MED/MSF), that exergy efficiency increases with increasing salinity. Note that the heat requirement increases with salinity; however, as the thermodynamic minimum also increases, whether the exergy efficiency will increase or decrease is not obvious.

The temperature differences associated with polarization phenomena, $\Delta T_{TP}$ and $\Delta T_{CP}$, are a result of the geometry of the flow channel and cross-flow velocity, and are generally proportional to the filtration flux (and thus $\Delta T_{D}$). The temperature difference over the heat exchanger ($\Delta T_{HX}$) is proportional to the amount of heat transported and depends implicitly on its geometry, dimensions, and operational conditions. For a given heat exchanger, the temperature difference between the feed and the permeate is also proportional to the flux or net driving temperature difference.

At this point, we neglect the additional boiling point elevation due to concentration polarization because it is minimal compared with the other temperature differences [24]. We assume that the conductive heat flow through the temperature polarization layer is much larger than the convective heat flow [20]. Furthermore, we also assume that the polarization phenomena are identical on both sides of the membrane. However, we can follow the same approach by replacing, for example, $2\Delta T_{TP}$ with $\Delta T_{TP,F}+\Delta T_{TP,P}$, etc. When the heat flow through the boundary layer is equal to the heat flow through the membrane, it follows that

(10) $$k_{BL}\Delta T_{TP}=\Delta H_{v}J_{W}+k_{M}\left(\Delta T_{D}+\Delta T_{B}\right)$$

and

(11) $$A_{HX}k_{HX}\Delta T_{HX}=A_{M}\Delta H_{v}J_{W}+A_{M}k_{M}\left(\Delta T_{D}+\Delta T_{B}\right)$$

Analogously to the membrane parameter, we can define a temperature polarization parameter and heat exchanger parameter, relating their conductive heat resistance to the evaporative heat transfer resistance of the membrane, as follows:

(12) $$K_{TP}=\frac{k_{TP}}{\Delta H_{v}L_{T}}$$

and

(13) $$K_{HX}=\frac{A_{HX}k_{HX}}{A_{M}\Delta H_{v}L_{T}}$$

After a substitution of the permeability (Equation (6)), membrane coefficient (Equation (8)), and the temperature polarization parameter (Equation (12)), into Equation (10), we find that

(14) $$\Delta T_{TP}=\Delta T_{D}\left(\frac{K_{T}+1}{K_{TP}}\right)+\frac{K_{T}}{K_{TP}}\Delta T_{B}$$

and

(15) $$\Delta T_{HX}=\Delta T_{D}\left(\frac{K_{T}+1}{K_{HX}}\right)+\frac{K_{T}}{K_{HX}}\Delta T_{B}$$

The substitution in Equation (9) and rearrangement yields

(16) $$\eta_{E}\eta_{D}={\left(1+2\frac{K_{T}}{K_{TP}}+2\frac{K_{T}}{K_{HX}}+\frac{\Delta T_{D}}{\Delta T_{B}}\left(1+2\frac{K_{T}+1}{K_{TP}}+2\frac{K_{T}+1}{K_{HX}}\right)\right)}^{-1}{\left(1+K_{T}\left(1+\frac{\Delta T_{B}}{\Delta T_{D}}\right)\right)}^{-1}$$

The driving temperature difference that minimizes the energy requirement ($\Delta T_{D}^{*}$) can be found by setting the derivative of Equation (17) with respect to $\Delta T_{D}$ to zero (to verify this step, we recommend the use of a symbolic mathematics software, such as Maple or Mathematica, as the process is rather tedious). After some rearrangement, this results in

(17) $$\frac{\Delta T_{D}^{*}}{\Delta T_{B}}={\left(\frac{K_{T}}{1+K_{T}}\right)}^{\frac{1}{2}}{\left(1+2\frac{K_{T}+1}{K_{TP}}+2\frac{K_{T}+1}{K_{HX}}\right)}^{-\frac{1}{2}}{\left(1+2\frac{K_{T}}{K_{TP}}+2\frac{K_{T}}{K_{HX}}\right)}^{\frac{1}{2}}$$

In this equation, two extreme cases can be considered. Firstly, if the heat transfer in the boundary layer can be neglected ($K_{TP},K_{HX}\gg K_{T}$), an upper bound is found. Secondly, if the heat transfer in the boundary layers is limiting ($K_{HX}\ll K_{T}$ and/or $K_{TP}\ll K_{T}$), then a lower bound is found:

(18) $$\frac{K_{T}}{K_{T}+1}\leq \;\frac{\Delta T_{D}^{*}}{\Delta T_{B}}\leq \sqrt{\frac{K_{T}}{K_{T}+1}}$$

Note that in this framework, the efficiency of a single effect only depends on the choice of the net driving temperature relative to the boiling point elevation and three dimensionless parameters, which describes the ratio of the conductive heat transfer and the evaporative heat transfer through the membrane.

Regarding the heat exchanger, we can also assume that it is dimensioned in a way that fixes its temperature difference ($\Delta T_{HX}$) (see reference [21], for example). This implies that the size of the heat exchanger is proportional to the net driving temperature difference in the membrane element. Following the procedure outlined above, we find that

(19) $$\eta_{E}\eta_{D}={\left(1+2\frac{K_{T}}{K_{TP}}+\frac{2\Delta T_{HX}}{\Delta T_{B}}+\frac{\Delta T_{D}}{\Delta T_{B}}\left(1+2\frac{K_{T}+1}{K_{TP}}\right)\right)}^{-1}{\left(1+K_{T}\left(1+\frac{\Delta T_{B}}{\Delta T_{D}}\right)\right)}^{-1}$$

and

(20) $$\frac{\Delta T_{D}^{*}}{\Delta T_{B}}={\left(\frac{K_{T}}{1+K_{T}}\right)}^{\frac{1}{2}}{\left(1+2\frac{K_{T}+1}{K_{TP}}\right)}^{-\frac{1}{2}}{\left(1+2\frac{K_{T}}{K_{TP}}+2\frac{\Delta T_{HX}}{\Delta T_{B}}\right)}^{\frac{1}{2}}$$

In this case, the dimensionless constant that represents the heat exchanger is the ratio of the temperature difference over the heat exchanger boundary layer and the boiling point elevation.

## 3. Results

### 3.1. Minimum Net Driving Temperature Difference for a Single Effect

**Figure 2:**
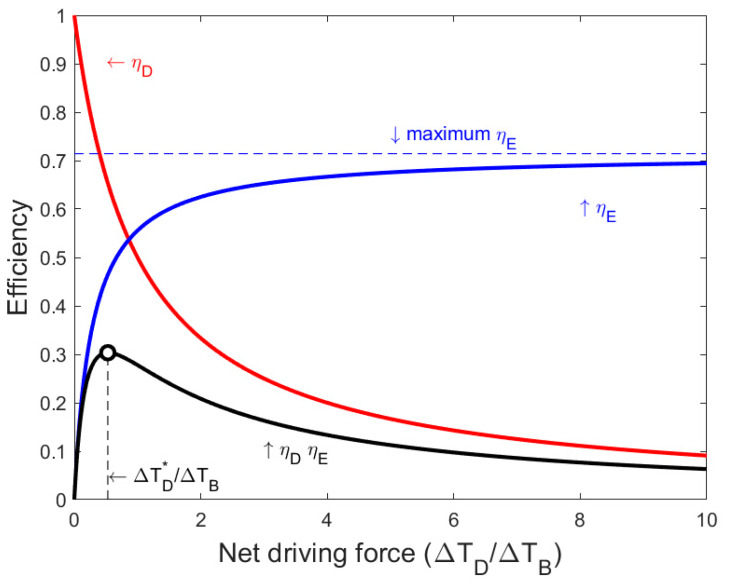
The trade-off between evaporation efficiency and driving force efficiency for a single effect, with negligible heat transfer resistance in the boundary layers near the membrane and the heat exchanger, and a membrane parameter of *K_T_* = 0.4. Evaporation efficiency increases with the net driving temperature, reaching a plateau at 1/(1 + *K_T_*). Driving force efficiency decreases with the net driving temperature. The product of these two efficiencies has a maximum.

Figure 2 illustrates the trade-off between the evaporation efficiency and the driving force efficiency for a particular membrane with $K_{T}$ = 0.4 and negligible polarization phenomena ($K_{TP},K_{HX}\ll K_{T}$). Figure 2 shows that, with an increasing net driving temperature, the evaporation efficiency increases, while the driving force efficiency decreases. The overall efficiency of a single effect is equal to the product of the driving force efficiency and the evaporation efficiency, and this efficiency has a maximum.

**Figure 3:**
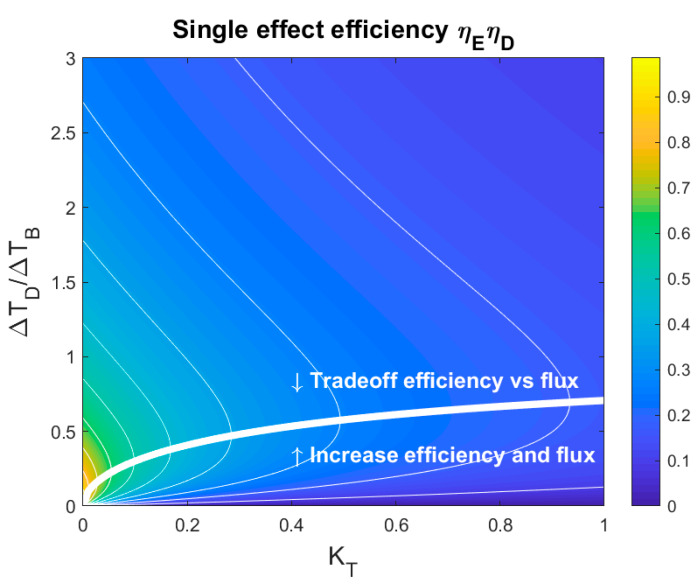
Single effect efficiency as a function of the membrane parameter (*K_T_*) and the net driving temperature, under the assumption that the heat-transfer resistance in the boundary layers is negligible (*K_TP_,K_HX_* ≫ *K_T_*). The white line shows the relation between the membrane parameter and the net driving temperature that maximizes the efficiency, and divides the area into two zones. In the lower zone, both the thermal efficiency and productivity may be improved by choosing a larger net driving temperature difference. In the upper zone, there is a trade-off between increasing the flux and lowering the thermal efficiency. A rational operational strategy will prefer an economically favorable trade-off between the heat consumption and productivity and thus will avoid the lower zone.

Figure 3 shows the efficiency as a function of the membrane parameter and the chosen net driving temperature. The previous figure (Figure 2) corresponds to a vertical line at $K_{T}=0.4$ in this plot, showing that the maximum efficiency occurs for every value of the membrane parameter. This maximum is represented in Figure 3 by the curve (Equation (17)) that divides the chart into two areas. In the lower area, both the efficiency and productivity can be improved by increasing the net driving temperature. In the upper area, there is a trade-off between improving the productivity and reducing the efficiency. A rational operational strategy will prefer a favorable trade-off between the heat requirement and production rate and thus will avoid the lower area. Thus, from an operational perspective, a net driving temperature higher than the net driving temperature difference that maximizes efficiency ($\Delta T_{D}^{*}$) should be chosen.

### 3.2. Influence of Heat Exchanger and Temperature Polarization

**Figure 4:**
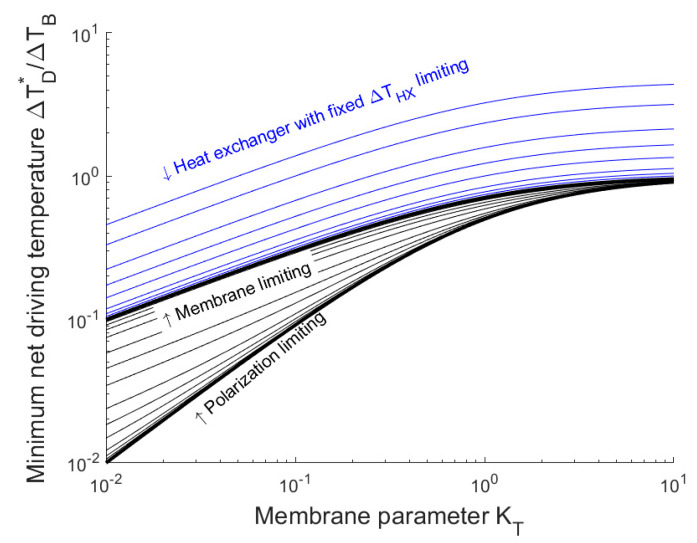
Minimum net driving temperature as a function of the membrane parameter, the heat exchanger parameter, and the temperature polarization parameter. The black lines correspond to the assumption that the temperature difference over the heat exchanger and temperature polarization is proportional to the net driving temperature, with various values of the parameter *K_HX_* and *K_TP_*. The thick lines correspond to the extreme scenarios outlined in Equation (17). The blue lines correspond to the assumption that the temperature difference over the heat exchanger is fixed, from top to bottom: Δ*T_HX_*/Δ*T_B_* = 10, 5, 2, 1, 0.5, etc.

Figure 4 shows the minimum net driving temperature as a function of the membrane parameter for several scenarios. First, we consider a scenario where the temperature difference over the heat exchanger is proportional to the net driving temperature (shown by the black lines in Figure 4), which occurs when the dimensions of the heat exchanger are fixed and the driving temperature is varied as an operational setting. We can see that for this scenario, the minimum driving temperature is less than the boiling point elevation and depends weakly on the heat transfer coefficient of the boundary layer or heat exchanger.

Next, we consider the scenario where the heat exchanger has a fixed temperature difference (the blue lines in Figure 4), which occurs when the size of the heat exchanger is varied, depending on the net driving temperature. In this scenario, we assume that the effect of *TP* is small compared with the effect of the heat exchanger. We can see that, in this case, the minimum net driving temperature is above the boiling point elevation, which depends strongly on the heat exchanger parameter and can become arbitrarily large. This suggests that in simulations and experiments where a fixed and relatively large value is chosen for $\Delta T_{HX}$, a relatively large value of the minimum net driving temperature may also be found.

### 3.3. Exergy Efficiency Limitation

**Figure 5:**
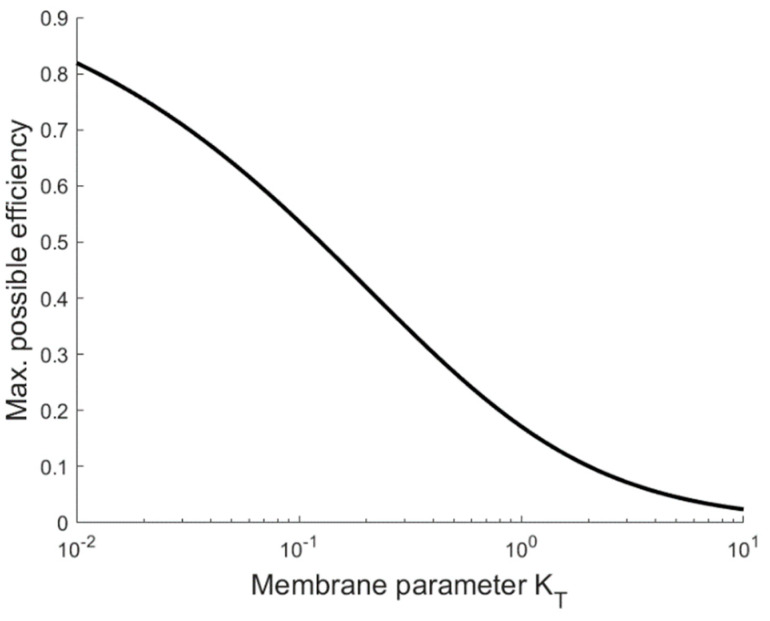
Exergy efficiency limitation as a function of the membrane parameter for a single effect from a multi-effect membrane distillation. The graph indicates the maximum thermodynamic efficiency that can be attained for a specific membrane if the polarization effects and the heat exchanger temperature difference are negligible.

The trade-off between the evaporation efficiency and driving force efficiency limits the maximum attainable exergy efficiency, even if a system can be designed in a way that the polarization phenomena in the boundary layer and near the heat exchanger are negligible. This exergy limitation is plotted in Figure 5. The relation between the membrane parameter and the maximum attainable efficiency makes it clear that understanding and reducing the membrane parameter is paramount for improving the energy efficiency of the MD process.

## 4. Discussion

### 4.1. Implications for Membrane Properties

Figure 5 shows that the membrane properties, represented by the membrane parameter, can severely limit the maximum attainable efficiency. The membrane parameter defined in Equation (8) equals the ratio of the permeability and the thermal conductivity. As is well established in the field, an optimal membrane that has a low thermal conductivity and high permeability will thus have a small value of $K_{T}$.

The membrane parameter can be reduced by maximizing the porosity [21]. Maximizing the porosity will increase the permeability and decrease the thermal conductivity under the reasonable assumption that the thermal conductivity coefficient of the membrane material is higher than the thermal conductivity coefficient of the gas in the pores [20,25]. With increasing porosity, the efficiency of MD is ultimately limited by the properties of the water vapor [3].

Note that both the conductivity and permeability depend inversely on the thickness of the membrane [20]. Consequently, the membrane thickness is canceled out in the ratio. Therefore, the membrane parameter, as well as the evaporation efficiency, are expected to be independent of the membrane thickness. Since the productivity is related to permeability, and thus inversely related to the membrane thickness, MD membranes should aim to be as thin as practically possible, considering, for example, manufacturing limitations, mechanical strength, and wetting.

The aim for a very thin MD membrane described above appears to contradict the results presented by Deshmukh et al. [21]. They investigated the effect of membrane thickness on thermal efficiency and found a membrane thickness that provides an optimal trade-off between the conductive heat loss and the required driving temperature difference. However, their result was obtained by setting a constant filtration flux while varying the thickness. As a consequence of this condition, they introduced a correlation between the thickness and the net driving temperature difference. Thus, we propose that in that study, in reality, they found an optimal temperature difference that coincided with a specific value of the thickness due to the condition of constant flux. We elaborate on this theory further in the following section.

### 4.2. Implicit Driving Temperature Variation

Here we apply the minimum driving temperature concept to the issue mentioned above concerning the thickness of the membrane. The variation in thickness with a constant flux corresponds to moving over a vertical line in Figure 3 because a smaller driving temperature is needed to maintain the flux while the membrane parameter remains constant. At some point, the minimum driving temperature is crossed (marked by the white line) and a further decrease in the membrane thickness negatively affects the heat requirement. Hence, for a different chosen filtration flux, a different optimal thickness would have been found. From an operational point of view, the flux is not required to be the same for each type of membrane. Thus, after reaching the minimum driving temperature, additional improvements in the permeability should translate into a higher filtration flux.

The minimum driving temperature concept can also be applied to understand other situations, where an optimum appears when the driving temperature is varied as a consequence of varying another parameter in combination with the applied constraints. For example, for a membrane with fixed properties, previous studies have shown that there is an optimal flux that maximizes the efficiency [21]. Below the optimal flux, both the productivity and energy consumption improve by increasing the flux, and there is a trade-off above the optimal flux. Another study found that the production capacity is inverted by increasing the number of stages (or length) of a DCMD system [26], and the inversion point coincided with a maximum efficiency. Thus, they found a zone where both the productivity and the heat requirement could be improved by increasing the length. The authors introduced the correlation between the driving temperature and the length by fixing the feed and permeate inlet temperatures.

### 4.3. Extension to More Complex Systems

When more complex systems are considered, it becomes important that the parameters ($K_{T}$, $K_{HX}$, and $K_{TP}$) depend on the temperature and that they have different values depending on the location. A similar approach to that outlined in this work can be applied, where an integral over the length of a membrane or a sum over multiple effects is minimized instead. Nevertheless, there must be a trade-off between the driving force efficiency, which prefers a low driving temperature, and the evaporation efficiency, which prerfers a large driving temperature difference. We anticipate that the optimal driving temperature for a single effect (Equations (17) or (20)) and the exergy limitation shown in Figure 5 can provide a reasonable estimate for the optimal average net driving temperature difference for more complex systems when the parameters are determined for the average temperature in the system $\left((T_{H}+T_{C}\right)/2)$.

## 5. Conclusions

We investigated the trade-off between the evaporation efficiency and driving force efficiency within a thermodynamic framework by following the heat flow for a single effect MD system, which provided the following conclusions:

- There is a non-zero net driving temperature difference that maximizes the thermodynamic efficiency of a single effect. Below this temperature difference, both the productivity and exergy consumption can improve by increasing the net driving temperature difference. Thus, this is the minimum net driving temperature difference that should be chosen for a rational operational strategy;
- In experiments where a particular parameter is varied while others remain fixed, the net driving temperature can be consequently varied. The minimum net driving temperature will then correspond to an “optimal” value of another parameter. Thus, we should be aware that, in these cases, the optimal value of the investigated parameter depends on the choice of the other fixed parameters;
- The minimum net driving temperature is proportional to the boiling point elevation (salinity) and is fully determined from three dimensionless constants, which are the ratio of heat transfer coefficients vs. the membrane permeability. Therefore, the treatment of very saline water is relatively more attractive, as this allows a reasonable driving force without incurring a substantial efficiency penalty.
